# Non-invasive Potential Circulating mRNA Markers for Colorectal Adenoma Using Targeted Sequencing

**DOI:** 10.1038/s41598-019-49445-x

**Published:** 2019-09-10

**Authors:** Vivian W. Xue, Moon T. Cheung, Pak T. Chan, Lewis L. Y. Luk, Vivian H. Lee, Thomas C. Au, Allen C. Yu, William C. S. Cho, Hin Fung Andy Tsang, Amanda K. Chan, S. C. Cesar Wong

**Affiliations:** 1 Department of Health Technology and Informatics, Faculty of Health and Social Sciences, Hong Kong Polytechnic University, Kowloon, Hong Kong China; 2 Department of Surgery, Queen Elizabeth Hospital, Kowloon, Hong Kong China; 3 State Key Laboratory in Oncology in South China, Sir YK Pao Centre for Cancer, Department of Clinical Oncology, Hong Kong Cancer Institute and Li Ka Shing Institute of Health Sciences, The Chinese University of Hong Kong, Sha Tin, Hong Kong China; 4 Department of Computer Science, University of Oxford, Oxford, United Kingdom; 5 Department of Clinical Oncology, Queen Elizabeth Hospital, Kowloon, Hong Kong China; 6 Department of Pathology, Queen Elizabeth Hospital, Kowloon, Hong Kong China

**Keywords:** Diagnostic markers, Cancer

## Abstract

We have developed an optimized protocol for plasma targeted mRNA sequencing in our previous study. Here, we performed plasma targeted mRNA sequencing for 40 colorectal adenoma patients and 39 colonoscopy-proven normal controls in order to find potential circulating mRNA markers for colorectal adenoma. Results showed that *GSK3A* and *RHOA* were differential expressed genes identified by a cut-off of fold change >2 and adjusted *P* value < 0.05. More detailed analysis showed that the expression of both *GSK3A* (0.01-fold with adjusted *P* < 1 × 10^−6^) and *RHOA* (0.35-fold with adjusted *P* < 0.01) in adenoma patients was significantly lower than those in normal healthy subjects. Based on the enrichment analysis of biological process for potential markers, we found that the regulation of programmed cell death (GO: 0043067; GO: 0043069), regulation of cell death (GO: 0010941; GO: 0060548) and cell differentiation (GO: 0021861) were the main processes involved in adenoma formation. In summary, this study is a cutting-edge research on the detection of plasma mRNA in colorectal adenoma patients and normal healthy subjects.

## Introduction

An early detection for colorectal adenoma is warranted because colorectal adenoma is the precancerous lesion of colorectal cancer (CRC). The removal of colorectal adenoma can prevent the development of CRC effectively. Moreover, the five-year survival rate of CRC patients in localized cases can approach 90%, which is significantly higher than those with distant metastasis (about 14%)^1^. This evidence shows the significance of an effective screening test for colorectal adenoma in clinical practice. Unfortunately, current screening strategies have limitations and still cannot achieve an effective early non-invasive diagnosis of colorectal adenoma. On one hand, faecal immunohistochemical tests (FIT) are not sensitive and specific enough in colorectal adenoma detection because adenoma rarely leads to intestinal bleeding^2,3^, although faecal tests are affordable and flexible for screening in general populations^4,5^. On the other hand, endoscopy is effective for detection of colorectal adenoma, but invasive procedures, which impose extra risks such as bleeding and perforation^6^. Besides, high costing and uncomfortable bowel preparation possibly lead to low compliance rates. There are about one-third of eligible people that has never participated in any screening for colorectal adenoma in the United States^7^. On the contrary, recent reports have shown that non-invasive screening strategies such as cell free circulating biomarkers are acceptable and feasible as high-throughput molecular technology has rapid development in the past decade^3,8^. Therefore, there is an urgent need for novel strategies to achieve non-invasive detection of colorectal adenoma.

In this study, we measured and compared the circulating mRNA concentrations in plasma samples from colorectal adenoma patients and normal healthy controls using targeted mRNA sequencing. We custom designed a “CRC-associated targeted RNA-Seq panel” (Supplementary Table 1) that combine three mRNA categories of (a) 8 identified circulating mRNA markers for CRC, (b) 6 mRNAs that are dysregulated in CRC tissues as validated by large cohort studies, and (c) mRNAs encoding the members of the Wnt pathway. Besides, the biological processes and molecular pathways possibly involved in colorectal adenoma formation based on sequencing data were illustrated. Last but not least, our sequencing data showed that subtle differences in the cycle number of PCR amplification in sequencing libraries preparation affect gene expression analysis. This study has discovered potential plasma mRNA markers for non-invasive detection of colorectal adenoma, and it has established a solid foundation to validate those potential biomarkers in a larger cohort of patients in future.

## Results

### Plasma total RNA concentration and quality

**Figure 1:**
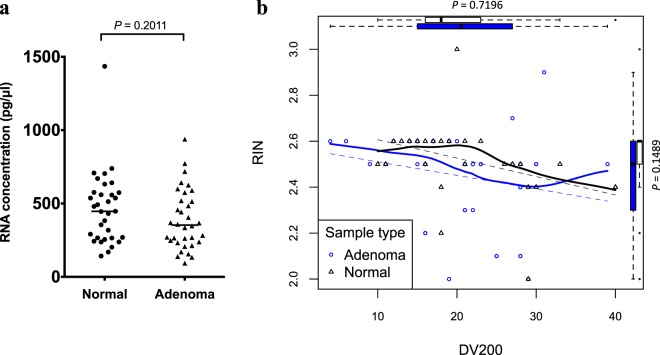
Plasma total RNA concentration and quality. The plasma total RNA **(a)** concentration and two parameters **(b)** RIN and DV_200_ for describing RNA quality had no significant difference in normal and adenoma plasma samples (*P* > 0.05, Mann-Whitney test). The similar relationships between RIN and DV_200_ in normal and colorectal adenoma plasma were observed. The solid line in **(b)** represented the best-fit curve calculated by Lowess function, and the dotted line represented the linear-fit result. The box and whisker plots at the top and the right side of **(b)** demonstrated the value distribution of DV_200_ and RIN, respectively.

Using bioanalyzer, RNA concentration was detected with median concentration of 447.0 (range: 142–1435) and 352.5 (range: 93–938) pg/μl in normal and adenoma plasma samples, respectively. For indicators of plasma RNA quality, RNA integrity number (RIN) was detected with median of 2.6 (range: 2–3) and 2.5 (range: 2–2.9) in normal and adenoma plasma samples, respectively. The percentage of RNA fragments >200 nt (DV_200_) was detected with median percentage of 18.0 (range: 10–40) and 20.5 (range: 4–39) in normal and adenoma plasma samples, respectively. The difference of RNA concentration and quality in normal and adenoma plasma samples was not statistically significant (Fig. 1). For 12 samples quantified by reverse transcription-quantitative polymerase chain reaction (RT-qPCR), details were shown in Supplementary Data 1.

### Summary of plasma mRNA targeted sequencing

Overall, the number of total raw reads in targeted sequencing on MiSeq was 153.1 million with at least 85.5% ≥ Q30. Among them, about 122.3 million reads were high quality based on MiSeq threshold, and 13.2% of them were aligned to the region of panel genes in human genome hg19. Raw reads, high quality reads, aligned reads and the ratio of alignment had no significant difference in colorectal adenoma samples compared with normal samples. Besides, there was no significant correlation between sequencing depth and DV_200_ (Spearman *r* = −0.200, *P* > 0.05) (Supplementary Fig. 1). Based on raw counts of RNA-Seq, 5 out of 108 (4.6%) genes were undetectable in both groups. Besides, 11 genes only could be detected in normal but not in colorectal adenoma plasma samples, while 5 genes only could be detected in colorectal adenoma but not in normal plasma samples. The summary of sequencing and gene detection were listed in Table 1, and “detectable” gene means that the gene was detectable in at least one sample.

**Table 1:** The summary of sequencing (median [range]) and gene detection (gene number [percentage]).

	Normal (*n* = 39)	Adenoma (*n* = 40)
**Summary of sequencing**		
Raw reads	1,548,980 (29,137–5,348,945)	1,741,836 (87,832–4,593,581)
High quality reads	1,264,269 (19,423–4,485,884)	1,386,364 (76,018–3,843,081)
Aligned reads (raw counts)	117,461 (3,130–879,796)	119,784 (1,532–934,219)
Alignment% (aligned reads/high quality reads × 100%)	13.5% (0.1–74.8%)	13.1% (0.1–72.9%)
***Summary of gene detection***		
Gene (*n* = 108)		
Detectable	98 (90.8%)	92 (85.2%)
In both groups	87 (80.6%)	87 (80.6%)
Only in this group	11 (10.2%)	5 (4.6%)
Undetectable	10 (9.2%)	16 (14.8%)
In both groups	5 (4.6%)	5 (4.6%)
Only in this group	5 (4.6%)	11 (10.2%)

### Overview of sequencing data

Firstly, 103 genes remained after removing genes with no expression in all samples. It was clear that genes undetectable in all samples provided no information about gene expression, and removing these genes from datasets had no effect on results^9^.

**Figure 2:**
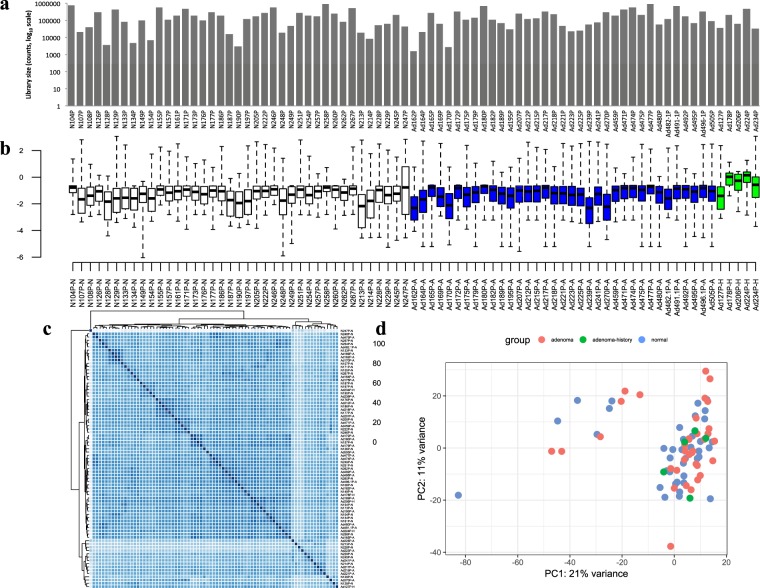
Overview of sequencing data. **(a)** Library size of each sample was showed as log_10_ scale. **(b)** Cook’s distance of adenoma and adenoma with malignant history samples was highlighted in blue and green color, respectively. Sample distances visualized by **(c)** clustering based on Euclidean distance and **(d)** PCA showed that plasma samples were not distinct based on their group labels. “N”, “A” and “H” labels of sample name indicate normal, adenoma and adenoma with cancer history groups, respectively.

For sequencing data, library size (log_10_ scale) and Cook’s distance of each sample were shown in Fig. 2a,b, respectively. There was no significant difference in sequencing library size of samples from normal, adenoma and adenoma with cancer history groups. However, Cook’s distance of adenoma samples with cancer history were slightly higher than those of both normal and adenoma samples. Cook’s distance is a parameter that describes how well a single sample will affect the fitness of generalized linear model. In Fig. 2b, normal and colorectal adenoma samples had comparable Cook’s distance, but 4 out of 5 colorectal adenoma samples with cancer history showed slightly higher Cook’s distance than other samples. To avoid potential effects on model fitness from colorectal adenoma samples with cancer history, they were analyzed independently from other adenoma samples in subsequent differential expression analysis.

The similarity of samples was visualized by the clustering based on Euclidean distance (Fig. 2c) and principal components analysis (PCA) (Fig. 2d). Our results showed that plasma samples were not distinct in expression profile based on their group labels. Additionally, normal plasma sample (lab no. 247) and adenoma plasma sample (lab no. 270) had different expression patterns compared with other samples, which was demonstrated by the PCA result. The differential expression pattern of normal plasma sample (lab no. 247) was easily distinguished by the clustering result. In PCA of sequencing data, all plasma samples were divided into two clusters, and two clusters were diagonally separated in Fig. 2d. The upper-left 12 samples (6 normal and 6 colorectal adenoma) were from the same sequencing run. In that run, sequencing libraries of all 12 samples were amplified using 33 PCR cycles. It was different in expression profile that all remaining samples at the lower-right corner were amplified using 35 PCR cycles in sequencing library preparation. This result illustrated the impact of different sequencing library preparation on sequencing results.

### Differential expression in normal and colorectal adenoma

Differential expression (DE) analysis and fold-change estimation of all 103 detectable genes were achieved by dispersion estimate and generalized linear model fitness in DESeq. 2. Results were listed in Table 2, and genes with significant difference in expression were highlighted in bold. *Glycogen synthase kinase 3 alpha (GSK3A)* and *ras homolog family member A (RHOA)* were two differential expressed genes (DEGs) detected between normal and colorectal adenoma based on the cut-off of fold change >2 and adjusted *P* value < 0.05. All DEGs met the adjusted *P* value < 0.1 were listed in Table 2, and the position information of detected sites of those genes was given. Both *GSK3A* and *RHOA* showed a significantly lower expression in plasma samples from colorectal adenoma patients as compared to normal controls. The expression of *GSK3A* in colorectal adenoma was significantly lower than those in normal plasma samples (0.01-fold with adjusted *P* < 1 × 10^−6^). *GSK3A* was detected in 22/39 (56.4%) normal plasma samples and 14/35 (40%) colorectal adenoma plasma samples with the median normalized expression of 8.0 (range: 0.0–57307.0) and 0.0 (range: 0.0–31591.0) CPM in normal and colorectal adenoma, respectively. The expression of *RHOA* in colorectal adenoma was significantly lower than those in normal plasma samples (0.35-fold with adjusted *P* < 0.05). *RHOA* was detected in all of normal and colorectal adenoma plasma samples with the median normalized expression of 147689.4 (range: 2341.2–936329.4) and 167443.6 (range: 2409.3–490713.5) CPM in normal and colorectal adenoma, respectively.

**Table 2:** Differential expressed genes analyzed by DESeq. 2 (Colorectal adenoma vs. Normal controls).

Gene ID	Chr	Start	Stop	Fold Change	*P* Value	Adjusted *P* Value
***GSK3A***	**19q13.2**	**42746373**	**42744257**	**0.01**	**2.20 × 10** ^**−09**^	**1.96 × 10** ^**−07**^
***RHOA***	**3p21.31**	**49412899**	**49405938**	**0.35**	**3.10 × 10** ^**−05**^	**1.38 × 10** ^**−03**^
*KRT19*	17q21.2	39684112	39681488	0.07	2.73 × 10^−03^	6.21 × 10^−02^
*TCF7*	5q31.1	133473825	133474683	0.22	2.79 × 10^−03^	6.21 × 10^−02^
*LEF1*	4q25	109086283	109084819	0.15	3.86 × 10^−03^	6.87 × 10^−02^
*MAPK8*	10q11.22	49609793	49612930	0.16	5.25 × 10^−03^	7.79 × 10^−02^
*BIRC5*	17q25.3	76212830	76219586	0.04	7.80 × 10^−03^	9.92 × 10^−02^

Statistical measures showed that the sensitivity and specificity of *GSK3A* mRNA were 90% and 38.5%, respectively whereas the positive and negative predictive values were 44.1% and 87.7%, respectively. For *RHOA* mRNA, the sensitivity and specificity were 70% and 31%, respectively whereas the positive and negative predictive values were 35.3% and 65.7%, respectively. Besides, there were no DEGs found in the comparison between normal and adenoma with cancer history and the comparison between adenoma and adenoma with cancer history.

**Figure 3:**
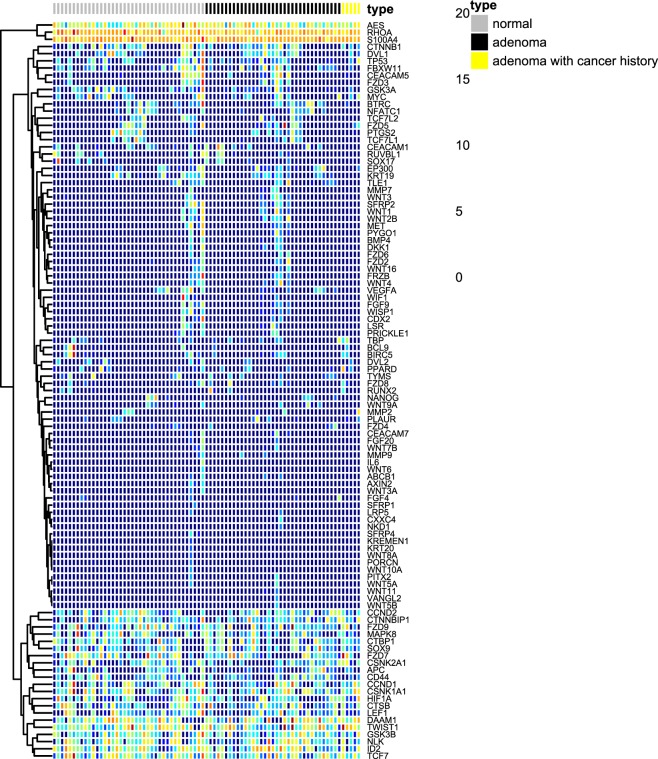
Hierarchical clustering of samples and gene expression in normal and colorectal adenoma. The hierarchical clustering of gene expressions in normal and colorectal adenoma plasma samples was achieved by complete linkage of Euclidean distance based on variance stabilizing transformed count data of 103 detectable genes in CRC-related mRNA panel.

The hierarchical clustering of variance stabilizing transformed count data was achieved by complete linkage of Euclidean distance (Fig. 3). Most of the genes in CRC-related mRNA panel in this study were low abundance in plasma samples, which was showed as navy blue area in Fig. 3. There were no gene clusters which showed an obviously differential expression between normal and colorectal adenoma samples.

## Discussion

In this study, we compared a panel of 108 CRC-related genes expression in plasma samples from colorectal adenoma patients and colonoscopy confirmed normal healthy subjects. Based on our knowledge, this is the first paper describing a targeted panel of plasma gene expression in colorectal adenoma patients. We focus on colorectal adenoma instead of CRC because not all colorectal adenoma will develop into CRC. Moreover, the prevalence of colorectal adenoma is much higher than CRC. Besides, the clinical outcome of colorectal adenoma is much less severe than that of CRC. Therefore it is important to perform targeted sequencing in the plasma of colorectal adenoma patients. We used the optimized protocol that we recently published in the Frontiers in Genetics, “The Effect of Centrifugal Force in Quantification of Colorectal Cancer Related mRNA in plasma Using Targeted Sequencing”^10^. Using targeted mRNA sequencing, we found that *GSK3A* and *RHOA* had significant difference in gene expression in colorectal adenoma patients as compared with normal healthy subjects (Table 2). It is worth noting that there was no statistically significant difference in the quality and quantity of total RNA between those 2 groups of subjects (Fig. 1). Statistical measures showed that *GSK3A* mRNA and *RHOA* mRNA had a much higher percentage of sensitivity than specificity which fulfils one of the requirements that a high sensitivity is preferred for screening and the results will have to be verified by conventional colonoscopy or sigmoidoscopy tests. In addition, their lower positive predictive values than negative predictive values showed that those genes are more reliable to rule out rather than to detect colorectal adenoma. The area under the curve (AUC) of *GSK3A* mRNA and *RHOA* mRNA were 0.629 and 0.508, respectively. As compared to those of CEA, faecal immunohistochemical and faecal DNA tests, their AUC values are 0.502^11^, 0.67^12^ and 0.73^12^, respectively. Therefore, the AUC values of *GSK3A* and *RHOA* showed that the performance of those 2 genes are not good enough for the detection of colorectal adenoma as compared to FIT and faecal DNA test. The main reason accounts for this is that this study is a preliminary trial and the sample size is not high enough to detect a small effect size of those 2 genes between pre-malignant colorectal adenoma patients and normal healthy individuals. Nevertheless, this is the first study to examine the feasibility of our custom designed gene panel and targeted sequencing protocol in the plasma of colorectal adenoma patients. Our preliminary results showed that *GSK3A* and *RHOA* were downregulated in the plasma of colorectal adenoma patients as compared to those of colonoscopy-proven normal subjects. The application of those 2 downregulated genes for colorectal adenoma detection in high risk patients and general population screening is difficult. In fact, we are planning to perform a large scale whole exome sequencing in order to find out potential up-regulated genes. Besides, the mechanisms which cause the downregulation of these 2 genes are being investigated so that we can verify our results and the mechanisms may be used as a potential marker for colorectal adenoma detection. Finally, the expression of those 2 genes would also be measured in CRC patients in order to examine whether a more significant down-regulation can be observed.

*Glycogen synthase kinase (GSK3)* has two isoforms *GSK3A* and *GSK3B*, and *GSK3B* shows increased expression in many types of cancer^13,14^. Compared with *GSK3B*, there is currently a lack of clear functional research on *GSK3A*. Previous study reported that *GSK3* isoforms had different expression and activity in prostate cancer^13^. Besides, *GSK3* isoforms worked in Alzheimer’s disease with distinct substrate preference^15^. However, these two isoforms showed mutually redundant in regulating drug-resistance in colon cancer cells^16^. *GSK3A* may be involved in immune response through regulating T lymphocytes functions or participating *CREB-GSK3A* signaling^17,18^. Reduced expression of *GSK3A* in colorectal adenoma may be the result of the dysfunction of immune response in tumorigenesis. *RHOA* was reported as a tumor suppressor gene in CRC^19,20^. The inactivation of *RHOA* promoted cancer cells invasion and de-differentiation by Wnt signaling pathway^20^. Specifically, the decreased expression of *RHOA* in CRC was probably caused by the copy number loss of this gene and its down-regulated transcription led by specific miRNA^19^.

**Figure 4:**
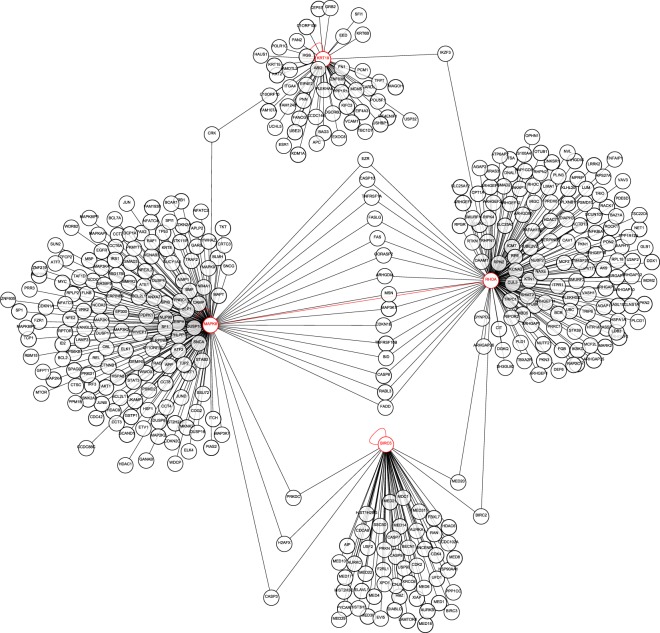
Enriched network of interactions among potential markers. Four genes (*RHOA*, *KRT19*, *MAPK8* and *BIRC5*) highlighted in red were potential markers found in this study for non-invasive and early diagnosis of colorectal adenoma. The solid line represented interactions, and the red line represented a direct interaction between two potential markers.

Meanwhile, other genes listed in Table 2 such as *keratin 19 (KRT19)* and *lymphoid enhancer binding factor 1 (LEF1)* could be true DEGs as well. However, the expression of these potential markers still needs to be confirmed by a thorough validation. For 7 potential markers for non-invasive and early diagnosis of colorectal adenoma listed in Table 2, enrichment analysis of biological process was performed. There were 53 significantly enriched gene ontology (GO) terms (FDR < 0.05, Fisher’s Exact with FDR multiple test correction), and the 10 most significantly enriched GO terms were shown in Table 3. Considering the custom CRC-related mRNA panel which was based on Wnt signaling pathway, it is expected that Wnt signaling pathway (GO: 0016055) was involved. Besides, regulation of programmed cell death (GO: 0043067; GO: 0043069), regulation of cell death (GO: 0010941; GO: 0060548), cell differentiation (GO: 0021861), apoptotic process (GO: 0043066) and cell-cell signaling (GO: 0198738) were also enriched biological processes. The significantly enriched network of interactions was shown in Fig. 4. Four of 7 potential markers (*RHOA*, *KRT19*, *MAPK8* and *BIRC5*) were involved. In detail, *KRT19* through *CRK proto-oncogene (CRK)* and *IKAROS family zinc finger 3 (IKZF3)* interacted with *MAPK8* and *RHOA*, respectively, and *BIRC5* through *caspase 3 (CASP3)*, *H2A histone family member X (H2AFX)*, *protein kinase (PRKDC)* and *baculoviral IAP repeat containing 2 (BIRC2)*, *mediator complex subunit 20 (MED20)* interacted with *MAPK8* and *RHOA*, respectively. However, there were no direct interactions between *KRT19* and *BIRC5*. For *MAPK8* and *RHOA*, they interacted with each other directly. Besides, there were several genes, such as *BH3 interacting domain death agonist (BID)* and *Fas cell surface death receptor (FAS)*, which mediated interactions between them.

**Table 3:** Enrichment analysis of biological process for potential markers.

GO biological process	Genes in term	Genes input	*P* value	FDR
Wnt signaling pathway (GO:0016055)	351	4	2.67 × 10^−06^	3.46 × 10^−03^
regulation of programmed cell death (GO:0043067)	1512	6	9.16 × 10^−07^	3.55 × 10^−03^
negative regulation of programmed cell death (GO:0043069)	867	5	2.36 × 10^−06^	3.67 × 10^−03^
negative regulation of apoptotic process (GO:0043066)	853	5	2.18 × 10^−06^	3.76 × 10^−03^
cell-cell signaling by wnt (GO:0198738)	351	4	2.67 × 10^−06^	3.78 × 10^−03^
somatic recombination of T cell receptor gene segments (GO:0002681)	5	2	1.99 × 10^−06^	3.86 × 10^−03^
negative regulation of cell death (GO:0060548)	946	5	3.63 × 10^−06^	4.02 × 10^−03^
forebrain radial glial cell differentiation (GO:0021861)	7	2	3.41 × 10^−06^	4.07 × 10^−03^
T cell receptor V(D)J recombination (GO:0033153)	5	2	1.99 × 10^−06^	4.41 × 10^−03^
regulation of cell death (GO:0010941)	1629	6	1.42 × 10^−06^	4.42 × 10^−03^

In addition, results generated from this study showed that targeted mRNA sequencing results were sensitive to different amplification protocols used in sequencing library preparation. In Fig. 2d, all plasma samples were divided into two clusters based on different amplification protocols (33 PCR cycles and 35 PCR cycles) instead of different biological types (normal and colorectal adenoma) by PCA. The same phenomenon could be observed in Fig. 3. The leftmost 12 samples showed a higher complexity of gene expression, and all of them were prepared by 33 PCR cycles amplification. This was likely due to increased cycles of amplification that allowed high-expressed genes to consume more sequencing capacity so that several low-abundance transcripts had not been detected^21^. Each amplification protocol involved in this study had its own flaws. Compared with the protocol using 35 PCR amplification cycles, the 33 PCR cycles protocol led to sequencing results with a higher complexity of gene expression. However, DEGs found by this protocol could not be validated by PCR-based methods due to their low expression level in plasma samples in some cases. For example, *WISP1* expression detected by targeted sequencing in our study was validated by RT-qPCR in plasma samples from another cohort of 8 healthy donors and 17 colorectal adenoma patients. The result showed that the expression of *WISP1* was only detected in 2 adenoma plasma samples with >40 Ct, and no expressions were detected in the remaining 23 plasma samples. When the result of qPCR > 40 Ct, it cannot exclude the possibility of a false-positive detection^22^. Therefore, the expression of *WISP1* detected by targeted mRNA sequencing in plasma samples using 33 PCR cycles protocol could not be validated by PCR-based methods. The validation of the targeted mRNA sequencing results of these low-abundance genes in plasma samples requires further studies using digital PCR, NanoString or other possible technological platforms. Besides, the performance of those potential biomarkers can be compared to carcinoembryonic antigen (CEA), a conventional marker for CRC.

Apart from circulating mRNA, microRNA (miRNA)^23^, circulating tumor DNA (ctDNA)^24^ and circulating tumor cell (CTC)^25^ also have potential in the non-invasive detection or screening of early stage cancers and precancerous lesions. In fact, they are more stable and abundant in plasma samples than circulating mRNA. Moreover, high throughput sequencing data of miRNA and ctDNA shows a better quality than that of circulating mRNA. However, mRNA markers can reflect sample information at transcriptome level. For the application of circulating mRNA markers, one of the promising ways is to integrate them with miRNA, ctDNA, methylated cell free DNA and CTCs in order to achieve multi-omics marker panels or screening strategies^26,27^. Tumorigenesis is a complex process involving crosstalk of multiple mechanisms, and many genes are mutually redundant in this process. Therefore, multi-omics marker panels or screening strategies can provide a more comprehensive cancer screening and monitoring strategy. Moreover, considering the effects of tumor heterogeneity and the vulnerable characteristics of circulating mRNA in the preservation and handling of liquid biopsy, it is a better choice to apply mRNA assay as a supplementary detection of early diagnosis compared to replacing current diagnostic strategies used in clinical applications such as endoscopy and computed tomography.

Up to now, there is only one study using high-depth small RNA sequencing in the plasma of colorectal adenoma patients^28^. This study is different from ours as they detected noncoding small RNAs, including microRNAs (miRNA), whereas we detected messenger RNA in the plasma of colorectal adenoma patients. In fact, our group has been working on the development of plasma messenger RNA using targeted sequencing in the plasma of colorectal cancer and adenoma patients^10^.

In conclusion, this study has laid down a solid foundation on the detection of plasma mRNA in colorectal adenoma patients and colonoscopy-proven normal subjects using this novel custom-design targeted gene panel and our established protocols. The results generated from this study can contribute to establish the mRNA-based non-invasive diagnosis of colorectal adenoma as a preventive strategy for CRC.

## Methods

### Patient recruitment and plasma collection

Forty colorectal adenoma patients and 39 normal healthy controls were recruited from the Department of Surgery, Queen Elizabeth Hospital, Hong Kong. Those normal controls were colonoscopy confirmed to be healthy. For each patient, anti-coagulated blood was collected by K3 EDTA tubes (Greiner Bio-one, Austria) and centrifuged for 1,600 *g*, 10 minutes at 4 °C. The clear upper layer plasma was collected and re-centrifuged for 16,000 *g*, 10 minutes at 4 °C to remove residual cell pellet. After that, 2.5 ml cell free plasma was collected and preserved by 2 ml Trizol Reagent (Thermo Fisher Scientific, USA) before storage at −80 °C. Blood processing was performed within 4 hours after blood draw.

Based on clinical records, 5 adenoma patients (lab no. 127, 178, 206, 224, 234) with adenocarcinoma history were excluded from adenoma group, and they were classified as a new group, namely adenoma with cancer history. Therefore, 39 normal controls, 35 colorectal adenomas and 5 adenomas with cancer history were included in the subsequent differential expression analysis as three groups respectively. All patients were histopathological confirmed their status, and detailed histopathological information of patients was shown in Supplementary Table 2. The study was conducted according to the principles expressed in the Declaration of Helsinki. Written informed consent was obtained from all patients and normal healthy controls. This study was approved by the Research Ethics Committee, Kowloon Central/Kowloon East, Hospital Authority, Hong Kong.

### Plasma RNA extraction and quality detection

For each sample, total RNA was extracted from 2.5 ml plasma. For every 4.5 ml plasma-Trizol Reagent mixture, 5.5 ml Trizol LS Reagent was added after mixture thawing to achieve the ratio of liquid sample:Trizol-Trizol LS mixture = 1:3 according to the manufacturer’s instructions. Total 10 ml mixture was mixed thoroughly and incubated 3 minutes at room temperature. The aqueous layer with RNA was separated after adding 2 ml chloroform (Sigma-Aldrich, USA) followed by centrifugation for 12,000 *g*, 15 minutes at 4 °C. Then, 1.5 volume of absolute ethanol (Sigma-Aldrich, USA) was added to the aqueous layer to achieve appropriate binding conditions.

The mixture was purified using miRNeasy Serum/Plasma Kit (Qiagen, Germany) according to the manufacturer’s instructions. Plasma total RNA was eluted in 14 μl RNase-free water and stored at −80 °C until use. Among all plasma samples, 6 normal and 6 colorectal adenoma plasma samples were done quality control by RT-qPCR, and the quality and quantity of RNA extracted from other plasma samples were detected using Agilent RNA 6000 Pico Kit on 2100 Bioanalyzer. RNA integrity number (RIN) and the percentage of RNA fragments >200 nt (DV_200_) were detected as the quality indicators.

### Sequencing library preparation and data analysis

Sequencing library was prepared using a custom designed TruSeq Targeted RNA Expression Kit (Illumina, USA) for detecting the expression of a panel of 108 CRC-related genes, and the panel of genes including 93 Wnt-signaling genes, CRC markers reported by previous studies and a control gene (Supplementary Table 1). The cDNA libraries were synthesized using 5 μl extracted plasma total RNA according to the manufacturer’s instructions with slight modifications, including (1) two-fold diluted adapters used in libraries PCR amplification; and (2) two times of clean-up for PCR products using AMPure XP beads (Beckman Coulter, USA)^29^. The quality and the quantity of prepared cDNA libraries were detected by Agilent High Sensitivity DNA Kit (Agilent Technologies, Lithuania) and qPCR, respectively (Supplementary Data 2). FastStart Universal SYBR Green Master (Roche, Germany) was used in quantification. Primers with 5′-AATGATACGGCGACCACCGAGAT-3′ and 5′-CAAGCAGAAGACGGCATACGA-3′ matched sequences within adapters were used. Illumina format DNA standard (Qiagen, Germany) was prepared by a serial dilution to achieve the standard curve for absolute quantification. Pooled sequencing libraries with 1% PhiX control (Illumina) were sequenced for single-end 51 bp length on MiSeq System using MiSeq Reagent Kit v3 (Illumina, Singapore).

Data analysis for targeted mRNA sequencing included two parts. The primary analysis was performed on MiSeq reporter. After base calling, FASTQ files of sequences with high sequencing quality were aligned and annotated based on indexes of custom designed regions on the hg19 reference genome. Raw counts of each gene for each sample were output as count matrix. The secondary analysis was performed by R 3.5.1^30^ and packages such as DESeq. 2, which were used to generate CPM (normalization) from count matrix, estimate dispersions, detect DEGs and visualize transcriptome profile based on RNA sequencing data^31^. The cut-off of fold change >2 and adjusted *P* value < 0.05 was used to identify significant differences. Adjusted *P* value was calculated based on Benjamini-Hochberg correction^32^. For selected DEGs, enrichment analysis was performed by GO, and biological processes with FDR < 0.05 (Fisher’s Exact with FDR multiple test correction) were identified as significantly enriched GO terms. The network of interactions among potential markers was analyzed using gProfiler.

### Statistical analysis

Statistical analysis for differential RNA concentration and quality, sequencing parameters (Table 1) was performed by Mann-Whitney test. The correlation of sequencing depth and DV_200_ was analyzed by Spearman correlation. Statistical analysis was performed in Prism 5 (GraphPad Software Inc, USA). *P* < 0.05 was regarded as significant difference. MedCalc statistical software version 18.9, MedCalc, Ostend, Belgium was used for ROC curves, sensitivity, specificity, positive and negative predictive values analysis.

## Supplementary information

Supplementary Figure S1

Supplementary Table S1

Supplementary Table S2

Supplementary Dataset S1

Supplementary Dataset S2
