# Supplementary material for: Non-invasive Potential Circulating mRNA Markers for Colorectal Adenoma Using Targeted Sequencing
Source: Sci Rep. 2019 Sep 10;9:12943. doi: 10.1038/s41598-019-49445-x (PMC6736954; doi:10.1038/s41598-019-49445-x)
Supplement: Supplementary file 1 — Supplementary Figure S1 [file 41598_2019_49445_MOESM1_ESM.pdf]

Non-invasive Potential Circulating mRNA Markers for Colorectal Adenoma Using Targeted Sequencing

Vivian W Xue[1], Moon T Cheung[2], Pak T Chan[2], Lewis LY Luk[2], Vivian H Lee[2], Thomas C Au[3], Allen C Yu[4], William CS Cho[5], Hin Fung Andy Tsang[1], Amanda K Chan[6], SC Cesar Wong[1,6*]

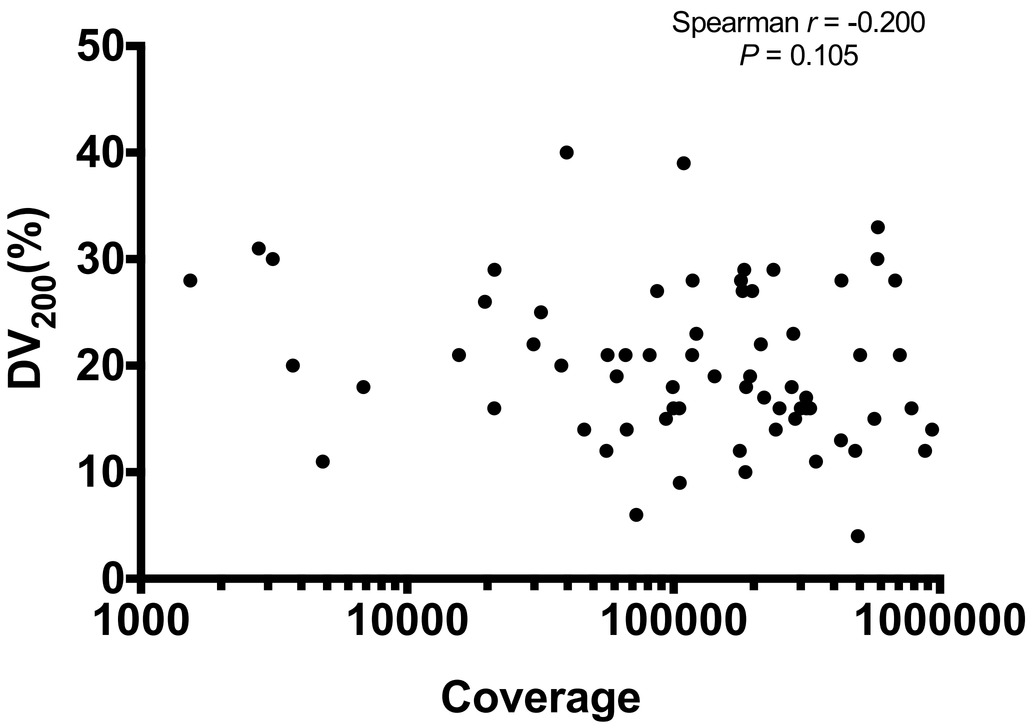

Supplementary Figure S1. The correlation of sequencing depth and DV_{200} was analyzed by Spearman correlation in Prism 5. $P < 0.05$ was regarded as significant correlation. There was no significant correlation between coverage depth and DV_{200} (Spearman $r = -0.200$, $P > 0.05$).
