# Supplementary material for: Non-invasive Potential Circulating mRNA Markers for Colorectal Adenoma Using Targeted Sequencing
Source: Sci Rep. 2019 Sep 10;9:12943. doi: 10.1038/s41598-019-49445-x (PMC6736954; doi:10.1038/s41598-019-49445-x)
Supplement: Supplementary file 3 — Supplementary Table S2 [file 41598_2019_49445_MOESM3_ESM.pdf]

Non-invasive Potential Circulating mRNA Markers for Colorectal Adenoma Using Targeted Sequencing

Vivian W Xue[1], Moon T Cheung[2], Pak T Chan[2], Lewis LY Luk[2], Vivian H Lee[2], Thomas C Au[3], Allen C Yu[4], William CS Cho[5], Hin Fung Andy Tsang[1], Amanda K Chan[6], SC Cesar Wong[1,6*]

Supplementary Table S2. The histopathological characteristics of patients (number {percentage})

Patients	Characteristics	Number (Percentage)
Normal (*n* = 39)		
	No record	31 (79.5%)
	Normal	8 (20.5%)
Adenoma (*n* = 40)		
Grade		
	Mild/Low grade of dysplasia	15 (37.5%)
	Moderate dysplasia	21 (52.5%)
	Severe/High grade of dysplasia	3 (7.5%)
	Unknown	1 (2.5%)
History of diagnosis		
	Adenocarcinoma	5 (12.5%)
	Adenoma	4 (10.0%)
	Polyp	1 (2.5%)
	Unknown	30 (75.0%)
Follow up diagnosis		
	Adenocarcinoma	2 (5.0%)
	Adenoma	6 (15.0%)
	Inflammation	1 (2.5%)
	Unknown	31 (77.5%)
