# Supplementary material for: Non-invasive Potential Circulating mRNA Markers for Colorectal Adenoma Using Targeted Sequencing
Source: Sci Rep. 2019 Sep 10;9:12943. doi: 10.1038/s41598-019-49445-x (PMC6736954; doi:10.1038/s41598-019-49445-x)
Supplement: Supplementary file 4 — Supplementary Dataset S1 [file 41598_2019_49445_MOESM4_ESM.pdf]

Non-invasive Potential Circulating mRNA Markers for Colorectal Adenoma Using Targeted Sequencing

Vivian W Xue[1], Moon T Cheung[2], Pak T Chan[2], Lewis LY Luk[2], Vivian H Lee[2], Thomas C Au[3], Allen C Yu[4], William CS Cho[5], Hin Fung Andy Tsang[1], Amanda K Chan[6], SC Cesar Wong[1,6*]

Supplementary Data S1

For 6 normal and 6 colorectal adenoma plasma samples have not been detected by Bioanalyzer, their plasma RNA concentration was measured by RT-qPCR.

In detail, total RNA extracted from buffy coat was used to do standard curve for RT-qPCR absolute quantification. The expression of housekeeping gene *GAPDH* was detected in these plasma samples for quantifying plasma RNA. The median concentration of plasma RNA was 106.3 (range: 58-774) and 224.0 (range: 101-499) pg/μl in normal and colorectal adenoma samples, respectively. TaqMan assay of *GAPDH* was shown as below.

Gene	Sequence	
GAPDH	Forward primer	ACATCGCTCAGACACCATG
	Probe	AAGGTCGGAGTCAACGGATTTGGTC
	Reverse primer	TGTAGTTGAGGTCAATGAAGGG
