# Supplementary material for: Non-invasive Potential Circulating mRNA Markers for Colorectal Adenoma Using Targeted Sequencing
Source: Sci Rep. 2019 Sep 10;9:12943. doi: 10.1038/s41598-019-49445-x (PMC6736954; doi:10.1038/s41598-019-49445-x)
Supplement: Supplementary file 5 — Supplementary Dataset S2 [file 41598_2019_49445_MOESM5_ESM.pdf]

# Non-invasive Potential Circulating mRNA Markers for Colorectal Adenoma Using Targeted Sequencing

Vivian W Xue[1], Moon T Cheung[2], Pak T Chan[2], Lewis LY Luk[2], Vivian H Lee[2], Thomas C Au[3], Allen C Yu[4], William CS Cho[5], Hin Fung Andy Tsang[1], Amanda K Chan[6], SC Cesar Wong[1,6*]

## Supplementary Data S2

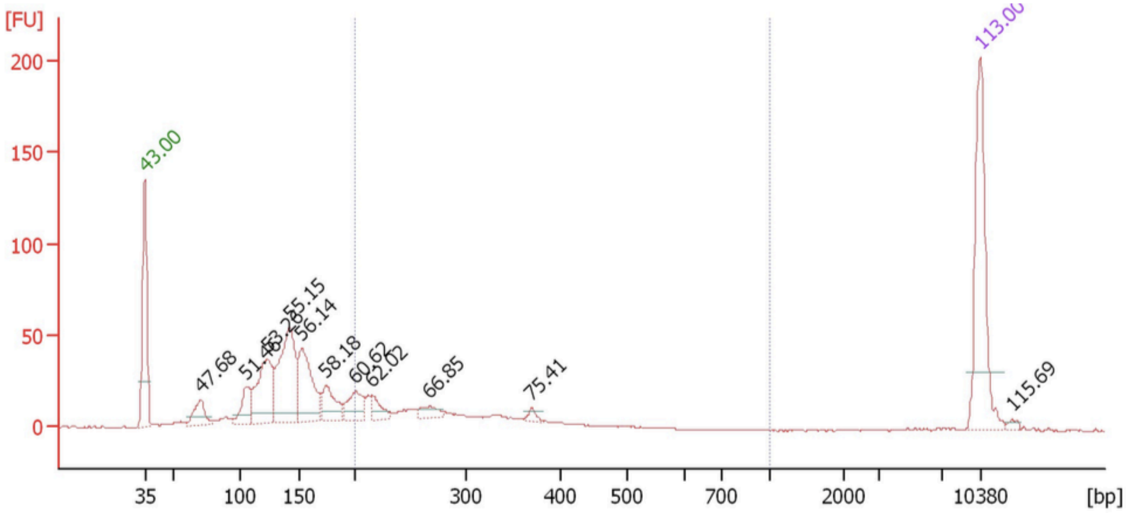


The fragment size of sequencing library was detected by 2100 Bioanalyzer, and it was shown above.

For the concentration of libraries quantified by qPCR, the median concentration of sequencing libraries prepared from normal and colorectal adenoma plasma samples were 0.43 and 0.45 nM/μl, respectively.
