# Supplementary material for: Non-invasive Potential Circulating mRNA Markers for Colorectal Adenoma Using Targeted Sequencing
Source: Sci Rep. 2019 Sep 10;9:12943. doi: 10.1038/s41598-019-49445-x (PMC6736954; doi:10.1038/s41598-019-49445-x)
Supplement: Supplementary file 2 — Supplementary Table S1 [file 41598_2019_49445_MOESM2_ESM.pdf]

Non-invasive Potential Circulating mRNA Markers for Colorectal Adenoma Using Targeted Sequencing

Vivian W Xue[1], Moon T Cheung[2], Pak T Chan[2], Lewis LY Luk[2], Vivian H Lee[2], Thomas C Au[3], Allen C Yu[4], William CS Cho[5], Hin Fung Andy Tsang[1], Amanda K Chan[6], SC Cesar Wong[1,6*]

Supplementary Table S1. CRC-related panel for targeted RNA sequencing

a)	Plasma CRC mRNA markers	CDX2	CTNNB1*	KRT19	KRT20	LSR	S100A4	TYMS	CEACAM1	CEACAM5
		CEACAM7								
b)	mRNAs dysregulated in CRC tissues	CCND1^	CDK8	PTGS2	CTSB	HIF1A	TP53			
c)	mRNAs of Wnt pathway (Illumina)	ABCB1	CD44	EP300	FZD5	LRP5	NLK	SFRP2	WIF1	WNT5B
		AES	CSNK1A1	FBXW11	FZD6	LRP6	PITX2	SFRP4	WISP1	WNT6
		APC	CSNK2A1	FGF20	FZD7	MAPK8	PLAUR	SOX17	WNT1	WNT7B
		AXIN1	CTBP1	FGF4	FZD8	MET	PORCN	SOX9	WNT10A	WNT8A
		AXIN2	CTNNB1*	FGF9	FZD9	MMP2	PPARD	TCF7	WNT11	WNT9A
		BCL9	CTNNBIP1	FOSL1	GSK3A	MMP7	PRICKLE1	TCF7L1	WNT16	
		BIRC5	CXXC4	FOXN1	GSK3B	MMP9	PYGO1	TCF7L2	WNT2B	
		BMP4	DAAM1	FRZB	ID2	MYC	RHOA	TLE1	WNT3	
		BTRC	DKK1	FZD2	IL6	NANOG	RUNX2	TWIST1	WNT3A	
		CCND1^	DVL1	FZD3	KREMEN1	NFATC1	RUVBL1	VANGL2	WNT4	
		CCND2	DVL2	FZD4	LEF1	NKD1	SFRP1	VEGFA	WNT5A	
d)	Control mRNA	TBP								

* shared between groups a) and c)

^ shared between groups b) and c)
